# Advanced Liquid Chromatography with Tandem Mass Spectrometry Method for Quantifying Glyphosate, Glufosinate, and Aminomethylphosphonic Acid Using Pre-Column Derivatization

**DOI:** 10.1021/acsestwater.3c00094

**Published:** 2023-05-24

**Authors:** Pedro J. Martin, Ke He, Lee Blaney, Shakira R. Hobbs

**Affiliations:** † Department of Civil & Environmental Engineering, Samueli School of Engineering, University of California, Irvine, Irvine, California 92697, United States; ‡ Department of Chemical, Biochemical, and Environmental Engineering, University of Maryland, Baltimore County, Baltimore, Maryland 21250-0001, United States

**Keywords:** reversed-phase chromatography, pesticide, micropollutant, glyphosate-based herbicides, pre-column derivatization, glufosinate, aminomethylphosphonic acid (AMPA)

## Abstract

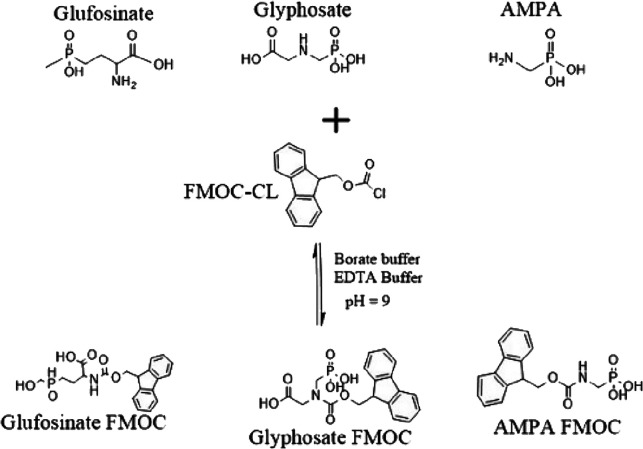

Analytical limitations make it challenging to develop effective methodologies for understanding glyphosate-based herbicide levels in drinking water and groundwater. Due to their lack of chromophores and zwitterionic nature, glyphosate-based herbicides are difficult to detect using traditional methods. This paper offers a straightforward method for quantifying glyphosate, glufosinate, and aminomethylphosphonic acid (AMPA) via 9-fluorenylmethylchloroformate (FMOC-Cl) pre-column derivatization and analysis by liquid chromatography with tandem mass spectrometry (LC–MS/MS). Method development was focused on optimizing the critical variables for optimal derivatization using a 2^4^-factorial design. We found that complete derivatization significantly depends on the inclusion of borate buffer to create the alkaline conditions necessary for aminolysis. Ethylenediaminetetraacetic acid (EDTA) addition was critical to minimize metallic chelation and ensure reproducible retention times and peaks. However, EDTA concentrations ≥5% decreased peak intensity due to ion suppression. The FMOC-Cl concentration and derivatization time exhibited a direct proportional relationship, with the complete reaction achieved with 2.5 mM FMOC-Cl after 4 h. Concentrations of FMOC-Cl greater than 2.5 mM led to the formation of oxides, which interfere with the detection sensitivity and selectivity. Desirable results were achieved with 1% EDTA, 5% borate, and 2.5 mM FMOC-Cl, which led to complete derivatization after 4 h.

## Abstract 2

The study presents a robust technique to detect glyphosate, enhancing comprehension of their effects on the environment and human health.

## 1 Introduction

The widespread occurrence of multiple pesticides in rivers and streams due to increased usage creates a complex exposure of compounds potentially toxic to environmental and public health.^1^ Glufosinate and glyphosate are broad-spectrum, nonselective, synthetic herbicides introduced in the 1970s for post-emergent weed management in several agricultural and non-crop applications.^2^ The use of glyphosate-based herbicides in agricultural operations has increased since the emergence of resistant plants, with an estimated yearly growth rate of 6.8% by 2024.^3,4^ The main metabolite, aminomethylphosphonic acid (AMPA), is most likely to be found because glyphosate has a short half-life.^5^ This breakdown product is frequently observed in surface waters and is produced by microbial organisms in soil and water.^4,6^

Recent studies indicated that herbicide residues have been discovered in sources of drinking water as a result of widespread and intensive use, which has an increasingly substantial negative influence on the environment,^5−9^ leading to the relevance and continued interest in routine monitoring of glyphosate, glufosinate, and AMPA. Several regulatory bodies are increasing the allowable residual limits after evaluating the carcinogenic hazard of glyphosate-based pesticides. In its regulatory investigations, the Environmental Protection Agency found that glyphosate generally has low toxicity to mammals. The World Health Organization nevertheless classified glyphosate as possibly carcinogenic to humans.^4,6,10^ As a result, there is controversy on the potential for toxicity and the optimal analytical approach to detect and quantify glyphosate, glufosinate, and AMPA in environmental matrices. The societal upheaval this problem has brought about has raised awareness of the discovery of glyphosate and AMPA in environmental samples. Appropriate methods to assess glyphosate, glufosinate, and AMPA at μg/L levels in aqueous samples are missing.^2,11,12^

Glyphosate-based compounds are commonly determined using chromatographic methods, such as reversed-phase and cation-exchange chromatography.^13−15^ Carboxylic phosphorylated polar herbicides like glyphosate have poor peak shapes and signals in mass spectrometry (MS) detectors due to poor ionization required for effective chromatographic separation. Metallic complexation also contributes to the poor peak shape during chromatography.^16^ Traditional technologies, such as gas chromatography (GC) and ion chromatography (IC), have demonstrated limitations in detecting the presence of glyphosate in water.^17^ Due to poor analytical reproducibility and sensitivity, glyphosate is difficult to measure due to complicated transitions in GC analysis.

Due to the existence of enantiomeric forms, some GC techniques detect glyphosate, glufosinate, and AMPA as multiple peaks, restricting quantitative measurement.^18^ While GC can relatively detect glyphosate and its derivatives in a sensitive and selective manner, the derivatization reaction produces unstable byproducts and is also very time-consuming. Polar organic micropollutants in water samples have been analyzed using ion chromatography with inductively coupled plasma (ICP) mass spectrometry. Although the detection limits were high, glyphosate and AMPA were identified in groundwater and surface water.^19^

New trends and interest in derivatized analyte detection using MS techniques have led to the development of efficient methodologies that combine liquid chromatography (LC) and MS.^20,21^ The use of liquid chromatography with tandem mass spectrometry (LC–MS/MS) improves the sensitivity and selectivity of detecting glyphosate, glufosinate, and AMPA in water. Chemical deprotonation allows for herbicide separation and retention on chromatographic columns.^22^ However, when employing this quantitative tool, there are numerous causes of uncertainty, including matrix effects, sample loss, physical and chemical interferences, and instrument detector drift. Detection via LC has reached limits of detection (LODs) as low as 20.0 μg/L, but to ensure fast, sensitive, and repeatable analysis of herbicides, derivatization processes and high-end equipment are required.^23^

Derivatization techniques have been employed in a number of studies to identify glyphosate-based molecules.^4^ Despite their benefits, these technologies are time-consuming and resource-intensive to operate. Pre- or post-column derivatization provides these compounds with chromophoric or fluorescent groups that enhance detection by conventional analytical instruments.^13,22,24,25^ Most pre-column techniques rely on derivatization with 9-fluorenylmethylchloroformate (FMOC-Cl) to produce ionizable derivatives that reduce polarity and aid chromatographic retention. Post-column derivatization shortcomings include the additional post-column dead volume required from large reaction loops, which reduce separation efficiency, and baseline variations caused by additional system noise from multiple delivery lines. Additional downsides include the need to regularly prepare samples for optimal sensitivity or keep them in an inert atmosphere to maintain their reactivity over time.^4,10^ For example, after chromatographic separation of the target compounds with a strong cation-exchange column, *o*-phthalaldehyde derivatization was utilized as a post-column derivatization since it is swift, but the derivatives are unstable after a few minutes.

In contrast, pre-column FMOC-Cl derivatization has been proven to be simple and successful.^14,21,22,26−28^ However, derivatization with FMOC-Cl is slower than with *o*-phthalaldehyde, and various reaction times have been proposed.^4,10^ There is some uncertainty due to the conflicting reports on the importance of reaction times for derivatization. The complete reaction of the glyphosate ion with FMOC-Cl guaranteed stability and successful chromatographic separation on LC columns.^10^ The derivatization period had a significant impact on issues related to acidification, buffer concentrations, and the generation of derivatization byproducts, such as FMOC-OH. The chromatographic analysis of glyphosate may be hampered by similar chromatographic transitions as the derivatives.^29,30^

Despite the gains in characterizing glyphosate in drinking water sources, more effort is needed to improve the robustness of detection techniques by eliminating false positives and matrix effects while determining trace levels. The objective of this study was to develop quantitative techniques to identify glyphosate, glufosinate, and AMPA by minimizing peak tailing and increasing retention times with sharper independent peaks to achieve lower detection limits. This research developed pre-column treatment techniques for LC–MS/MS detection of glyphosate-based herbicides at μg/L levels.

## 2 Materials and Methods

### 2.1 Reagents and Materials

Glyphosate (99%), AMPA (99%), and glufosinate-ammonium (99%) were obtained from Chemservice (West Chester, PA). Primary stock solutions containing 1 μg/μL concentrations for all chemicals (corrected for purity) were prepared in deionized (DI) water. A 100 μg/L working solution of glyphosate, glufosinate, and AMPA was prepared and stored at 4 °C. For sample fortification and calibration standards, several combined solutions of all target compounds ranging from 0.5 to 10 μg/L were created and employed as spike solutions. Isotopically labeled standards, 1,2-^13^C_2_^15^N glyphosate (99%) and ^13^C^15^N AMPA (99%), were obtained from Cambridge Isotope Laboratories (Andover, MA). The internal standard solution was made in DI water with a 50 μg/L concentration. LC–MS grade water, EDTA, acetonitrile (ACN), sodium tetraborate, FMOC-Cl, methanol (CH_3_OH), and phosphoric acid (H_3_PO_4_) were obtained from Fischer Scientific (Hampton, NH). A 0.1% phosphoric acid (v/v) in ACN/DI water (70:30) was used for rinsing the autosampler before and after injection.

### 2.2 Analytical Equipment and Conditions

The LC–MS/MS system included an Agilent Series 1290 LC and an Agilent 6470A triple quadrupole mass spectrometer (Agilent Technologies, Palo Alto, CA). The LC was equipped with a binary pump, column oven, ultraviolet detector, and autosampler. A Phenomenex Gemini NX-C18 column (3 μm particle size, 100 mm length, and 2.1 mm internal diameter) was used with a 0.4 mL/min solvent flow rate. The column compartment temperature was set to 40 °C, and the injection volume was 20 μL. Mobile phase A was 5 mM ammonium acetate in LC–MS grade water, and B was pure ACN. For the separation, the LC gradient was as follows: isocratic from 0 to 2 min (90% A, 10% B); linear increase of B from 10 to 25% for 3 min; linear increase of B from 25 to 50% for 3 min; linear decrease to 10% B for 1 min; and isocratic for 3 min (90% A, 10% B) to re-equilibrate the column to initial conditions. The method run time was 12 min. Following LC separation, negative mode electrospray ionization was used to introduce the analytes into the MS. The settings included a drying gas flow of 5 L/min, a drying gas temperature of 300 °C, a nebulizer pressure of 45 psi, and a 110 V fragmentor voltage. One precursor and one daughter ion were monitored for each compound, along with the retention times and mass/charge ratios. The mass transition ion pairs for the target ions are shown in Table 1.

**Table 1:** Summary of Multiple Reaction Monitoring Run Conditions, Including the Precursor and Daughter Ions^a^

FMOC-Cl compounds		PI	QI	CE
glufosinate FMOC-Cl	Q	402.1	180	8
	q	402.1	206	16
glyphosate FMOC-Cl	Q	389.9	168	12
	q	389.9	63	66
AMPA FMOC-Cl	Q	332.1	110	4
	q	332.1	136	14
isotope-labeled +4-AMPA FMOC-Cl	Q	336.0	114	4
isotope-labeled +3-glyphosate FMOC-Cl	Q	391.9	170	12

a PI, precursor ion; QI, quantitation daughter ion; CE, collision cell energy; Q, quantification transition; q, confirmatory transition.

### 2.3 Pre-Column Derivatization with FMOC-Cl

A 40 mL aliquot was pipetted into an amber glass bottle along with 800 μL of the internal standard solution, which was added to correct matrix effects. The pH of the matrix and standard solutions was adjusted to 9 by adding 2 mL of borate buffer, 2 mL of EDTA solution, and 6 mL of FMOC-Cl stock solution. The borate buffer ensured proper sample derivatization conditions, whereas the FMOC-Cl agent increased the molecular weight and stability of the analytes of interest for chromatographic separation. Metallic chelation was eliminated with the EDTA addition. Then, samples were placed in a water bath at 40 °C in the dark. To stop the derivatization, 2.4 mL of the phosphoric acid solution was added and kept at 4 °C. For effective chromatographic separation, the effects of the EDTA buffer strength, borate buffer strength, FMOC-Cl concentration, and derivatization time were investigated and optimized, as shown in Table 2.

**Table 2:** Investigated Factors That Affect the Effectiveness of FMOC-Cl Derivatization Glyphosate, Glufosinate, and AMPA

derivatization factor	varied values
time (h)	0, 1, 2, 4, 8, 24
borate (%w/v)	0, 1, 5, 10
EDTA (%w/v)	0, 1, 5, 10, 20
FMOC-Cl (mM)	1, 2.5, 5, 10, 20

### 2.4 Design of Experiments for Variable Optimization

**Figure 1:**
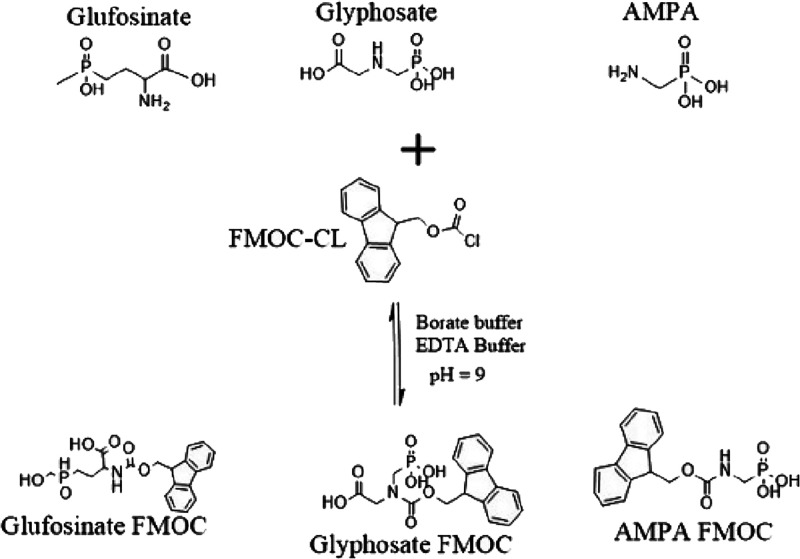
Derivatization reaction between glyphosate, glufosinate, AMPA, and FMOC-Cl.

A 2^4^ factorial design was employed to assess the effects and interactions of FMOC-Cl concentrations, derivatization times, borate buffer concentrations, and EDTA buffer concentrations for best-performing parameters in Table 2 (Figure 1). The best-performing variables from Table 2 were evaluated. JMP statistical software (Cary, NC) was used to build a desirability coefficient to discover which combinations of the different factors resulted in the most desirable outcomes. The four major effects and three two-way interactions were the focus of the desirability function’s experimental goal, which was to determine the settings of the variables to optimize the compound responses. The response variable is converted to a 0–1 scale via the desirability function. The reaction scale goes from 0 for the lowest response to 1 for the strongest response. The overall desirability is created by combining each individual response’s desirability using the geometric mean to concurrently maximize several responses.

### 2.5 Method Validation

The retention times, mass/charge ratios, and relative ion ratios were used to identify the target compounds. The linearity of the method was validated using seven standard solutions in triplicate. The LOD and limit of quantification (LOQ) were determined using various sample and standard spikes ranging from 1 to 10 μg/L in DI water. Spiked duplicates were employed to ensure accuracy and precision. The LOD and LOQ for each analyte were established as the lowest concentration that yielded signal-to-noise ratios (S/N) of 3 and 10, respectively, by gradually reducing the spiked analyte concentrations to achieve the closest values corresponding to those S/N ratios. Method repeatability and interday precision were determined by applying nested design, evaluating three replicates daily at low concentrations (10 μg/L) and three at high concentrations (100 μg/L) on three consecutive days.

## 3 Results and Discussion

### 3.1 Evaluation of FMOC-Cl Derivatization

#### 3.1.1 Effect of Borate Addition

**Figure 2:**
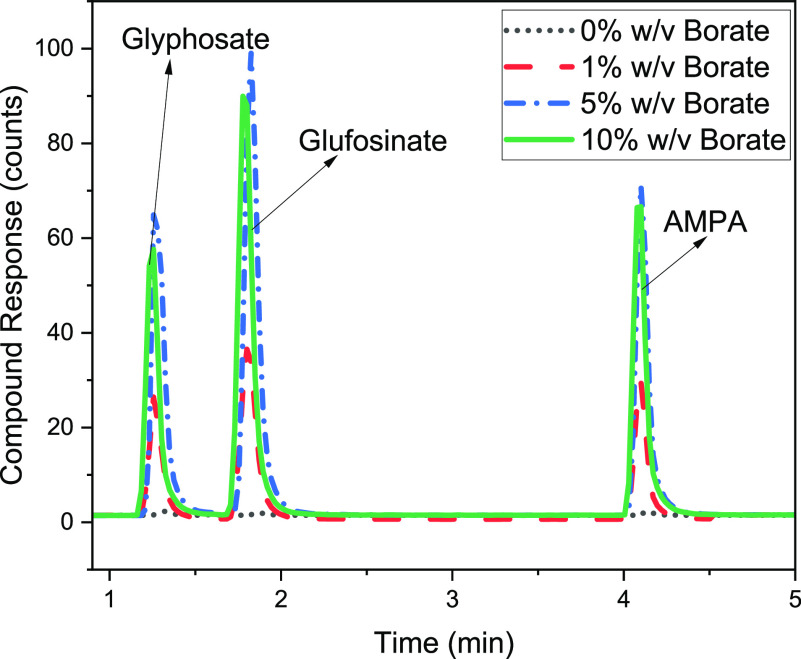
Chromatographic response of target compounds with varied borate concentrations.

Figure 2 highlights the response of the various borate buffer concentrations (w/v) tested to determine the best conditions: 0 (control), 1, 5, and 10%. The reaction did not occur in the control, for which no borate buffer was added. This performance can be attributed to the requirement of alkaline conditions necessary for complete aminolysis shown in Figure 1. When borate buffer was added to the reaction medium, the peak intensities of the derivative products were greater. The reactivity of glyphosate’s amino group was enhanced by increasing the buffer concentration, which improved the derivatization reagent’s solubility.^31−33^ The addition of 1% borate greatly improved the peak response of glyphosate, glufosinate, and AMPA compared to the control (Figure S1). However, when the concentration of the borate buffer was increased to 5 and 10%, a significant compound response was seen (*p* < 0.05). The correlation between the compound response and the reaction medium’s alkalinity was established as a result of the rise. However, a significantly higher response was obtained with 5% borate addition compared to the control than the other variables (*p* < 0.05). This observation underlines the requirement for borate to ensure full derivatization. The alkaline conditions created by the 5% borate buffer support Ehling and Reddy’s results about the optimum pH range of 8–10 as the concentration slightly increases the pH and reduces the compound response.^32^

#### 3.1.2 Effect of EDTA Addition

**Figure 3:**
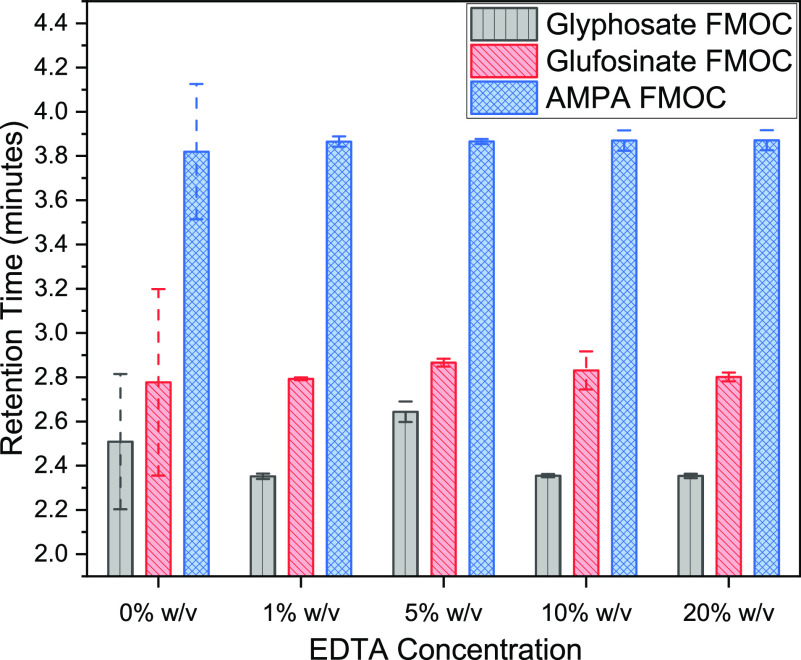
Highlights variation in retention times of target compounds with varied EDTA concentrations (*n* = 3, error bar = standard deviation).

**Figure 4:**
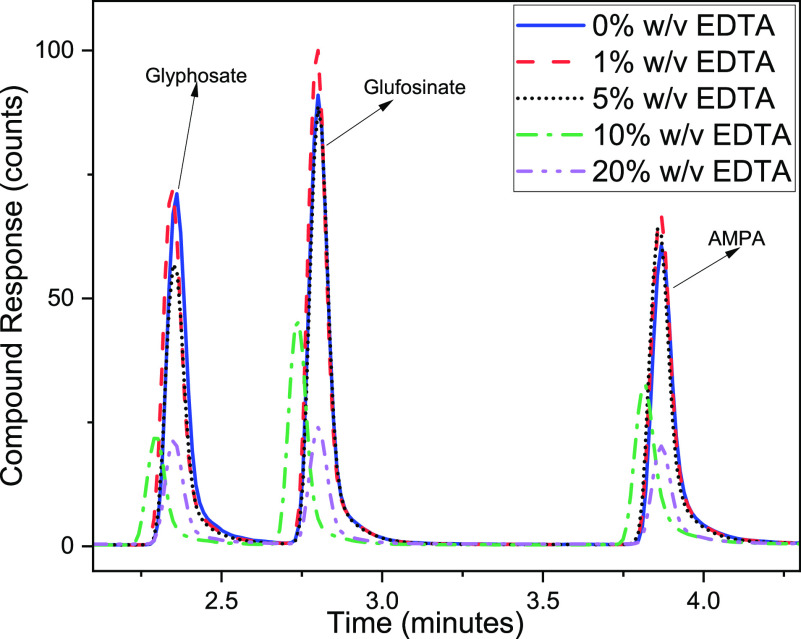
Highlights chromatographic response of target compounds with varied EDTA concentrations.

The performance of the EDTA buffer concentration was evaluated with 100 μg/L (each) standards of glyphosate, glufosinate, and AMPA (Figure 3). The control reaction without EDTA had a higher variation in analyte retention times (Figure S2). That variation was significantly reduced by introducing 1 and 5% EDTA buffer (*p* < 0.05). However, EDTA concentrations ≥5% reduced the abundance of all compounds compared to the control and 1% EDTA levels. Except for the 1% EDTA solution, all EDTA levels exhibited an adverse impact on abundance (Figure S3). For all three target compounds, the peak abundance with 1% EDTA was significantly higher than the control (*p* < 0.05) and 5% EDTA (*p* < 0.05) conditions. The addition of the EDTA solution minimized the shift in retention times in multiple runs and ensured stable peak abundance results (Figure S2). This trend was true for each of the three chemicals. The EDTA addition eliminated the poor intensity correlated to trace metal contamination in the LC–MS/MS system and enhanced detection of the polar phosphorylated herbicides (Figure 4).

In LC–MS investigations, it is frequently noted that phosphorylated chemicals and organic acids with numerous carboxylate groups produce poor peak shapes and signals. The phenomenon was also observed for glufosinate, AMPA, and glyphosate.^34^ The presence of trace metals, notably iron, contributed from several sources inside the chromatographic system is the reason for the poor peak shape. EDTA was used to address the trace metal contamination problem.^35^ EDTA, although a potent metal chelator, caused ion suppression on columns when retained for the higher concentrations >1%.^36^ Chelation decreases efficiency and symmetry due to the interaction between the mobile phases, additives, sources other than solute/stationary phase interfaces, and to various degrees depending on the instrument.^34^ The compound response in the 1% EDTA solution emphasizes the importance of removing poor peak shapes caused by multiple charged negative-ion analytes.

#### 3.1.3 Effect of FMOC-Cl Concentration

**Figure 5:**
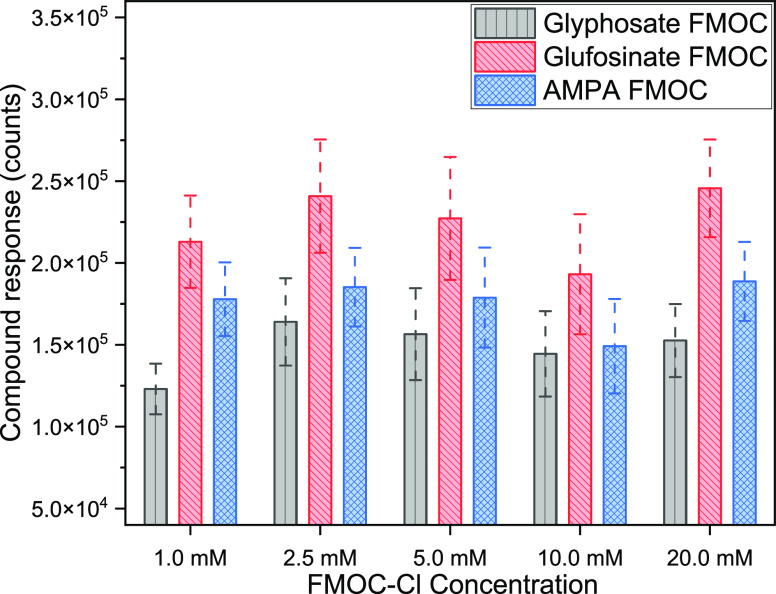
Effect of the FMOC concentration on the chromatographic response (*n* = 3, error bar = standard deviation).

Results demonstrated an improved FMOC-glyphosate area when the concentration of the derivatization reagent was raised from 1.0 to 2.5 mM (Figure 5). However, at the 5 and 10 mM FMOC-Cl concentrations, a negative effect was observed for the FMOC-glyphosate area (Figure S4). FMOC-glyphosate concentrations >2.5 and <20 mM experienced a considerable decrease in the chromatographic response. This adverse performance was attributed to the formation of oxides of the derivatization agent. The interaction between FMOC-Cl and water causes FMOC-OH to be produced at a higher concentration of the derivatization reagent.^11^ Because it is poorly soluble in water and has the potential to precipitate, this byproduct can hinder glyphosate detection. As a result, high FMOC-Cl concentrations affect chromatographic separation and reduce FMOC-glyphosate ionization.^30^ Even though precipitation was observed with addition of 20 mM FMOC-Cl, all target chemicals responded significantly better (*p* < 0.05). The excess FMOC-OH can be removed by solid-phase extraction.^8^

#### 3.1.4 Effect of Derivatization Time

**Figure 6:**
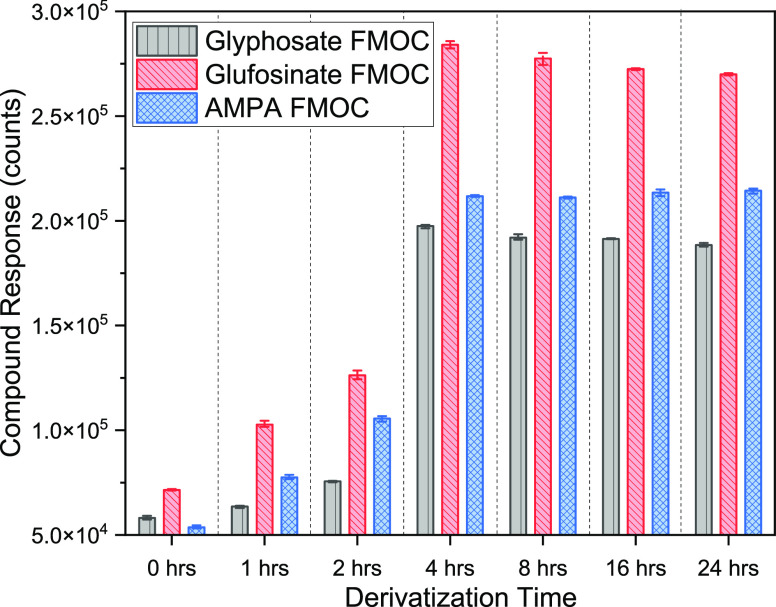
Effect of the time of derivatization on the chromatographic response (*n* = 3, error bar = standard deviation).

Figure 6 highlights the investigation into the impact of time on the derivatization process to establish the best time to convert the target molecule into MS ionized products. The length of derivatization ensured sufficient interaction of the analytes with the derivatizing reagent and complete conversion into identifiable ionized compounds. The reaction times required for the reaction of FMOC-Cl by glyphosate have been reported as 2–24 h for complete derivatization.^10^ It was however evident that the derivatized products were not stable before 4 h and showed any significant variations in the peak areas.^4^ Results demonstrated that the glyphosate, glufosinate, and AMPA derivative products peaked and remained stable after 4 h (Figure 6). Additionally, the derivative products did not exhibit any appreciable fluctuations in response for either, glyphosate, glufosinate, or AMPA. The minor decline in the glyphosate response for derivatization times after 4 h could be attributed to the conversion to AMPA as a relatively proportional increase is exhibited.^10^

### 3.2 Optimization of Derivatization Factors

**Figure 7:**
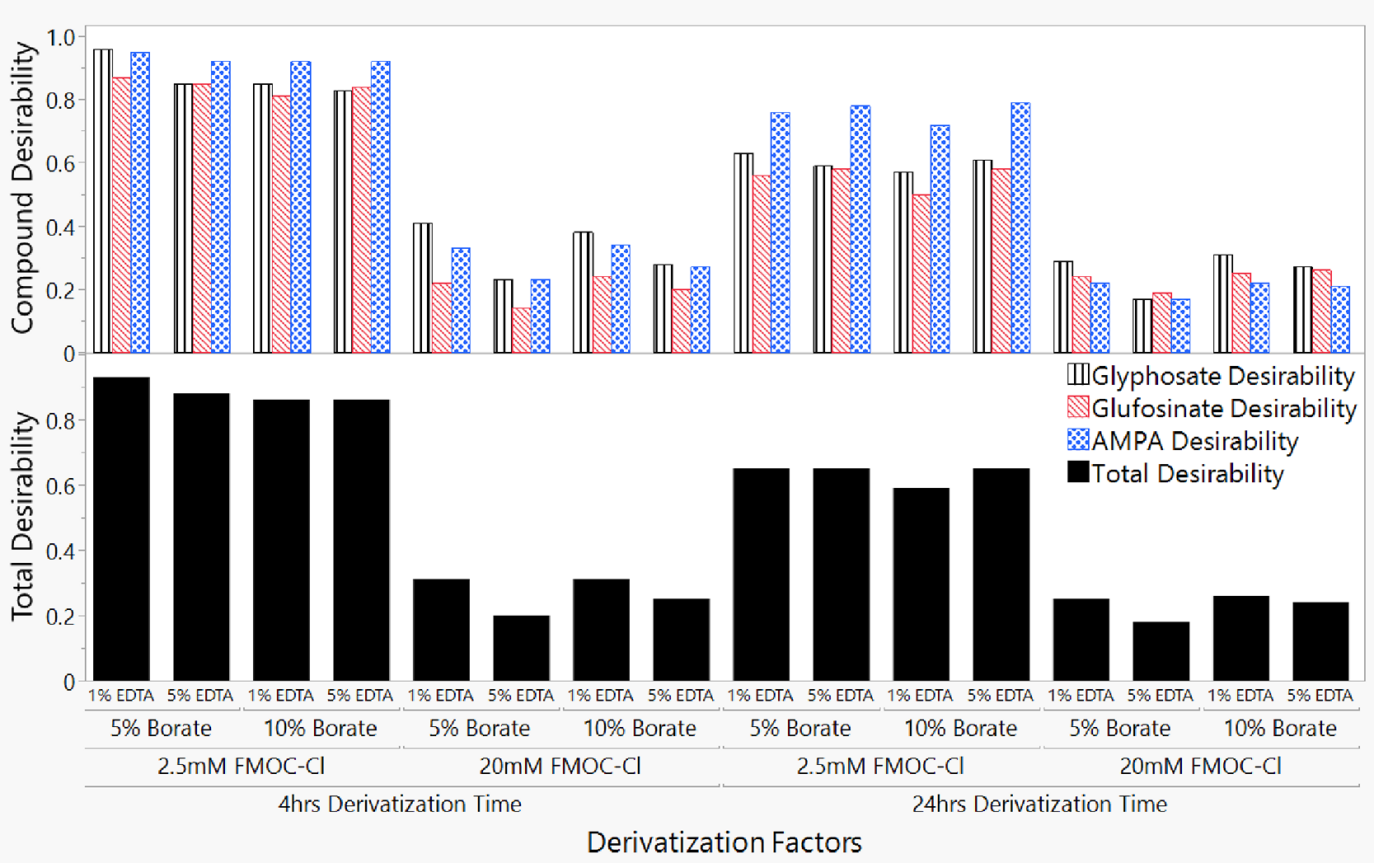
Desirability of optimal conditions for the complete derivatization of glyphosate, glufosinate, and AMPA in terms of reaction time and derivatizing agent concentration.

Figure 7 depicts the scenarios regarding the desirability of optimizing the derivatization factors through the interactions of the individual responses. The desirability function accounted for the simultaneous effect optimization for the top two performing alternatives after the individual response investigation. The optimization indicated that the optimal derivatization time for a complete reaction was 4 h, as most combinations of the other derivatization factors investigated performed best under the short derivatization time. All best-performing circumstances also involved the lower concentration of the derivatizing agent FMOC-Cl, whereas the higher concentration resulted in poor performance irrespective of time. The optimal derivatization occurs at 1% EDTA, 5% borate, and 2.5 mM FMOC-Cl after 4 h (Table S1). The interrelationship of the derivatization variables provided insight into the analysis of target chemicals for abundance in trace quantities while reducing background noise (Figure S6). The optimization evaluation improved the water analysis analytical technique by applying compound-specific stable isotopes.^11^ Although various variations of derivatization agent and times resulted in weaker performances (Figure 6), the addition of borate was critical to the effectiveness of FMOC-Cl derivatization of glyphosate, glufosinate, and AMPA (Figure 2). The interaction profiles for the various derivatization factors are highlighted in Figure S6. When the concentration of the EDTA was kept at 1%, all target compound responses were higher for the 5% borate addition than for the 10% alternative. However, when the EDTA concentration was 5%, the response for 10% borate was significantly higher than 5% borate (*p* < 0.05). There is an interaction between the concentration of the borate buffer and the time of derivatization, such that for the longer derivatization time, the 5% borate addition had a higher compound response than the 10% borate addition. However, the shorter derivatization time recorded a similar performance for both alternatives of the borate concentrations. This result indicates that a complete reactivity with the target compounds can be achieved in the shortest time possible with either borate concentration. Hence, 5% borate was selected for the optimized method. The interaction between borate and FMOC-Cl concentrations demonstrated that the 10% borate buffer performed better than the 5% borate buffer variable when the concentration of the derivatizing agent, FMOC-Cl, was 20 mM. The reverse effect was observed when the FMOC-Cl derivatizing agent was decreased; the 5% borate performed significantly higher than the 10% borate addition. This indicates a proportional relationship between the two factors and underlines the need for borate addition to ensure the complete derivatization of the target compounds.

## 4 Validation

The method performance parameters obtained for the pre-column derivatization procedure are shown in Table 3. Linearity was confirmed from the evaluation of the residual distribution, and coefficients of determination (*R*^2^) were ≥0.98 (Figures S7–S12). Statistical evaluation of the results showed that intercept values were not significantly different. Repeatability and interday precision, namely, relative standard deviation (RSD) <4.5% at the low level and <3.8% at the high level, were considered acceptable. No target compounds were detected in the blank controls. Table 3 also includes the LODs and LOQs. The method limits were obtained from instrumental limits using an analytical process, and as the S/N is an instrumental LOD, they do not significantly increase the variability and bias of analytical data. The outcomes show that the derivatization reaction significantly reduces the findings’ substantial variability, which must be considered. The quantification limits for the examination of waters are near the European drinking water quality regulations, which establish a parametric value for pesticides of 0.1 μg/L,^6^ a number that is frequently surpassed while monitoring surface waters. To obtain detections below the EU regulations and requirements for this application, a post-derivatization concentration and reconstitution process would be necessary, even though this has no bearing on the optimal derivatization conditions discovered in this work.

**Table 3:** Validation Parameters for the Optimized Derivatization Method

	precision (RSD, %) *n* = 3							
	repeatability		interday		limits (0.01–1 μg/L)		calibration (0.01–1 μg/L)	calibration (1–100 μg/L)
compounds	low	high	low	high	LOD	LOQ	*R*^2^	
glyphosate	3.50	2.10	3.70	0.60	0.04	0.12	0.989	0.997
glufosinate	1.20	0.70	1.10	0.10	0.03	0.09	0.994	0.997
AMPA	4.40	3.70	2.30	1.10	0.05	0.16	0.981	0.995

## 5 Conclusions

This study developed and optimized an accelerated and simplified method of quantifying glyphosate, glufosinate, and AMPA using FMOC-Cl derivatization. Although this matrix is complex and challenging, optimizing the chromatographic and experimental parameters produced a rapid, sensitive, and accurate assay. The assessed parameters included borate and EDTA buffer addition, FMOC-Cl concentration, and derivatization time. Complete reactivity with high sensitivity was achieved with 2.5 mM FMOC-Cl after 4 h. Higher concentrations of FMOC-Cl produced byproducts generated from the reaction between water and amino acids in sample matrices caused analytical interference and must be separated from the targeted analytes. The method was validated to meet all the requirements of selectivity, linearity, lower limit of quantitation, matrix effects, and stability. After derivatization, the optimized method, despite the complexity, was sufficiently sensitive and precise to quantify glyphosate and AMPA residues in water. The complexation and metallic interaction with target chemicals, varying retention times, and reduced glyphosate, glufosinate, and AMPA sample sensitivity were resolved. This work highlighted the required selectivity and sensitivity for the trace level measurement of glyphosate, glufosinate, and AMPA due to their ionic and polar characteristics.

## Supporting Information Available

Chromatographic responses for targeted compounds, interaction profiles, calibration curves for borate, EDTA, FMOC, and the interaction between various derivatization factors (PDF)
