# Supplementary material for: Advanced Liquid Chromatography with Tandem Mass Spectrometry Method for Quantifying Glyphosate, Glufosinate, and Aminomethylphosphonic Acid Using Pre-Column Derivatization
Source: ACS ES T Water. 2023 May 24;3(8):2407–14. doi: 10.1021/acsestwater.3c00094 (PMC10425981; doi:10.1021/acsestwater.3c00094)
Supplement: Supplementary file 1 — ew3c00094_si_001.pdf [file ew3c00094_si_001.pdf]

Advanced Liquid Chromatography with Tandem Mass Spectrometry Method for Quantifying Glyphosate, Glufosinate, and Aminomethylphosphonic Acid Using Pre-Column Derivatization

Pedro J. Martin [1], Ke He [2], Lee Blaney [2], and Shakira R. Hobbs [1]
[1]University of California, Irvine, Department of Civil & Environmental Engineering, Samueli School of Engineering, Irvine, California, USA.
[2]University of Maryland Baltimore County, Department of Chemical, Biochemical, and Environmental Engineering, Baltimore, USA.

Supporting Information

List of Figures

Supporting Information: Figure S1 - Chromatographic response of target compounds with varied borate concentrations S1
Supporting Information: Figure S2 - Highlights (a)variation in retention times of target compounds with no EDTA addition and (b-e) minimal variation with the addition of EDTA S2
Supporting Information: Figure S3 - Chromatographic response of target compounds with varied EDTA concentrations S3
Supporting Information: Figure S4 - Chromatographic response of target compounds with varied FMOC concentrations S4
Supporting Information: Figure S5 - Chromatographic average response of target compounds with varied derivatization times S5
Supporting Information: Figure S6a - Interaction profile of the various derivatization factors for Glyphosate S7
Supporting Information: Figure S6b - Interaction profile of the various derivatization factors for Glufosinate S8
Supporting Information: Figure S6c - Interaction profile of the various derivatization factors for AMPA S9
Supporting Information: Figure S7 - Calibration curve for Glufosinate (low level) S10
Supporting Information: Figure S8 - Calibration curve for AMPA (low level) S11
Supporting Information: Figure S9 - Calibration curve for Glyphosate (low level) S12
Supporting Information: Figure S10 - Calibration curve for Glufosinate (high level) S13
Supporting Information: Figure S11 - Calibration curve for AMPA (high level) S14
Supporting Information: Figure S12 - Calibration curve for Glyphosate (high level) S15

List of Tables

Supporting Information: Table S1 - Desirability of optimal conditions for the complete derivatization of glyphosate, glufosinate, and AMPA in terms of reaction time and derivatizing agent concentration S6

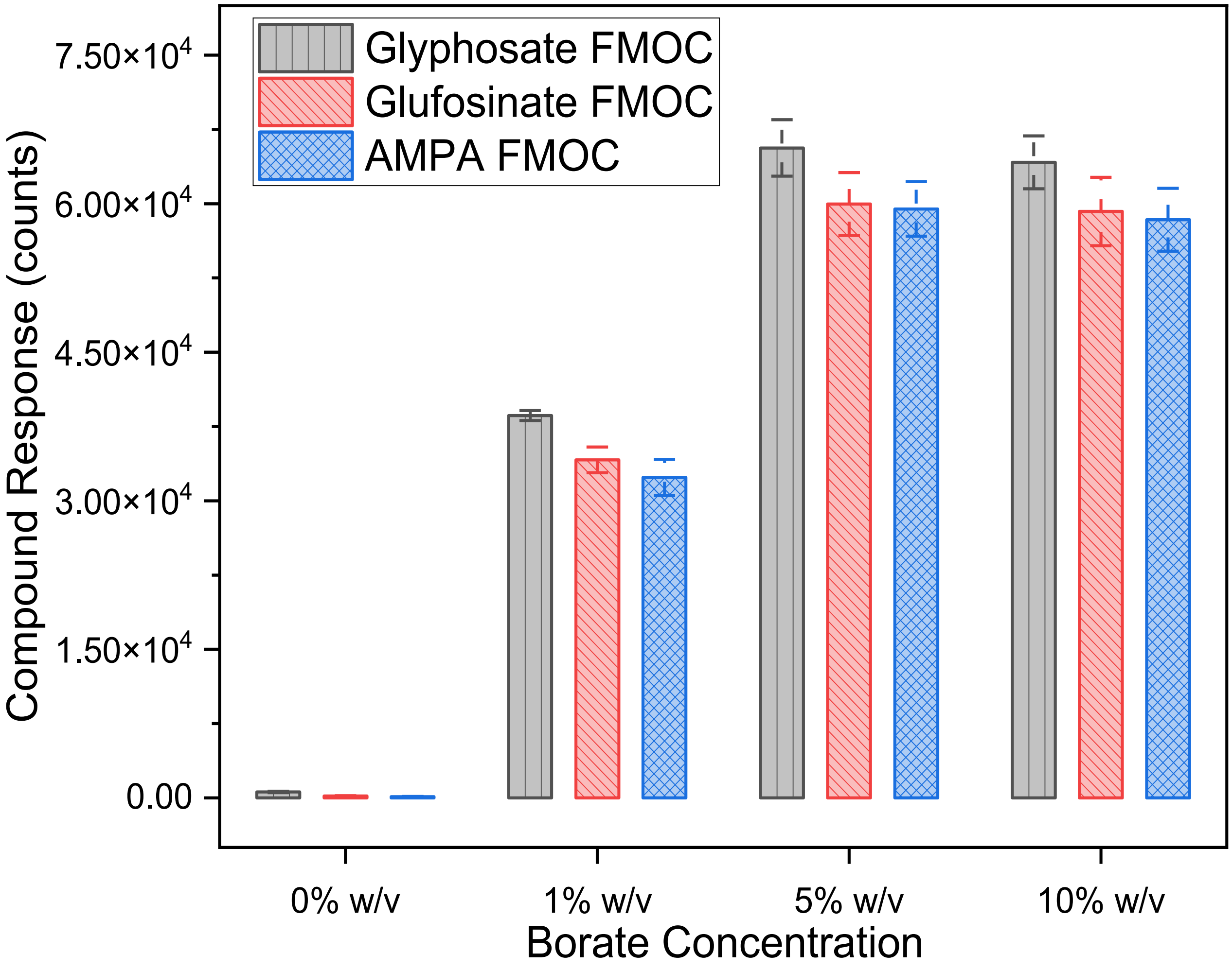

Supporting Information: Figure S1 - Chromatographic response of target compounds with varied borate concentrations (n=3, error bar = standard deviation).

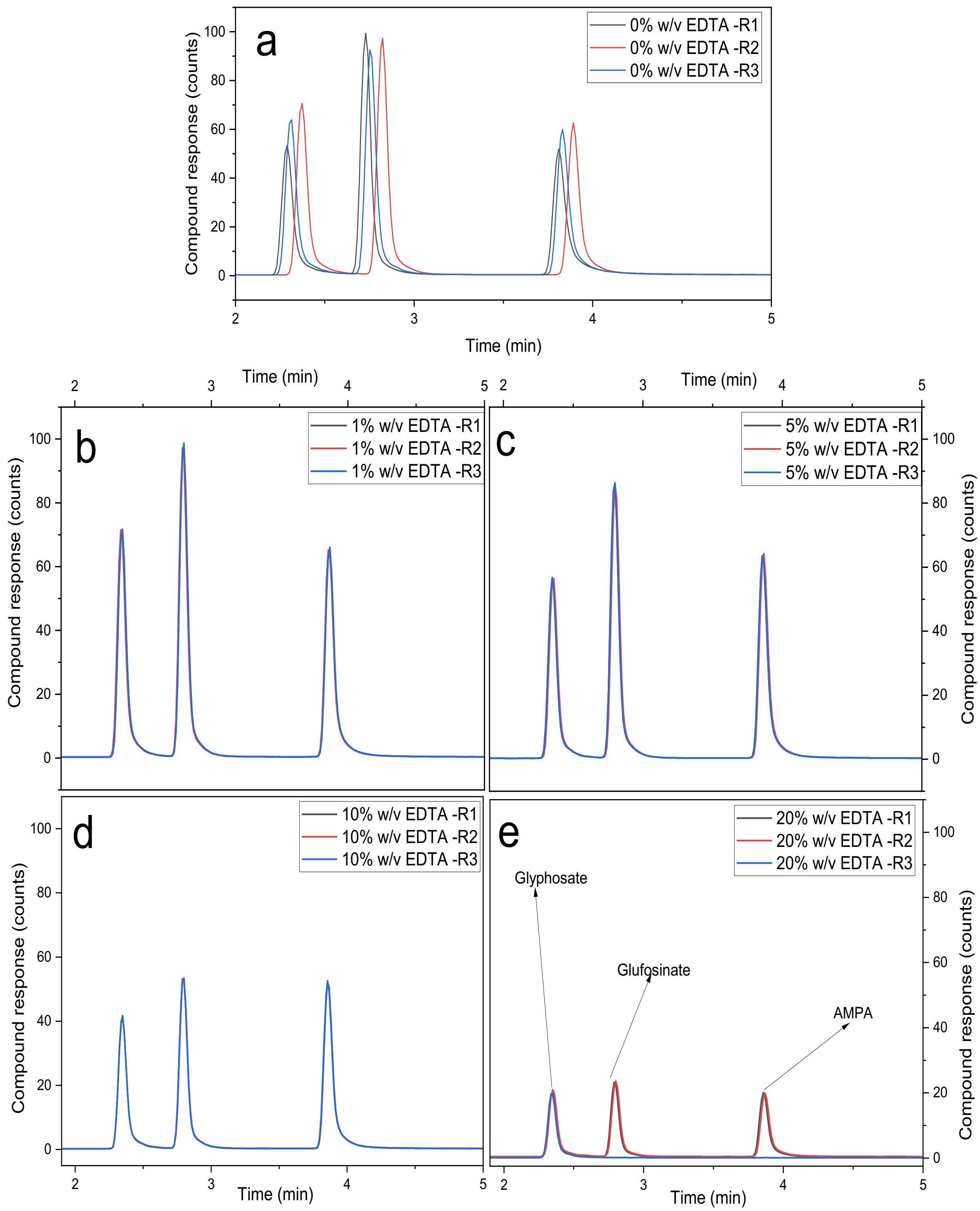

Supporting Information: Figure S2 - Highlights (a)variation in retention times of target compounds with no EDTA addition and (b-e) minimal variation with the addition of EDTA.

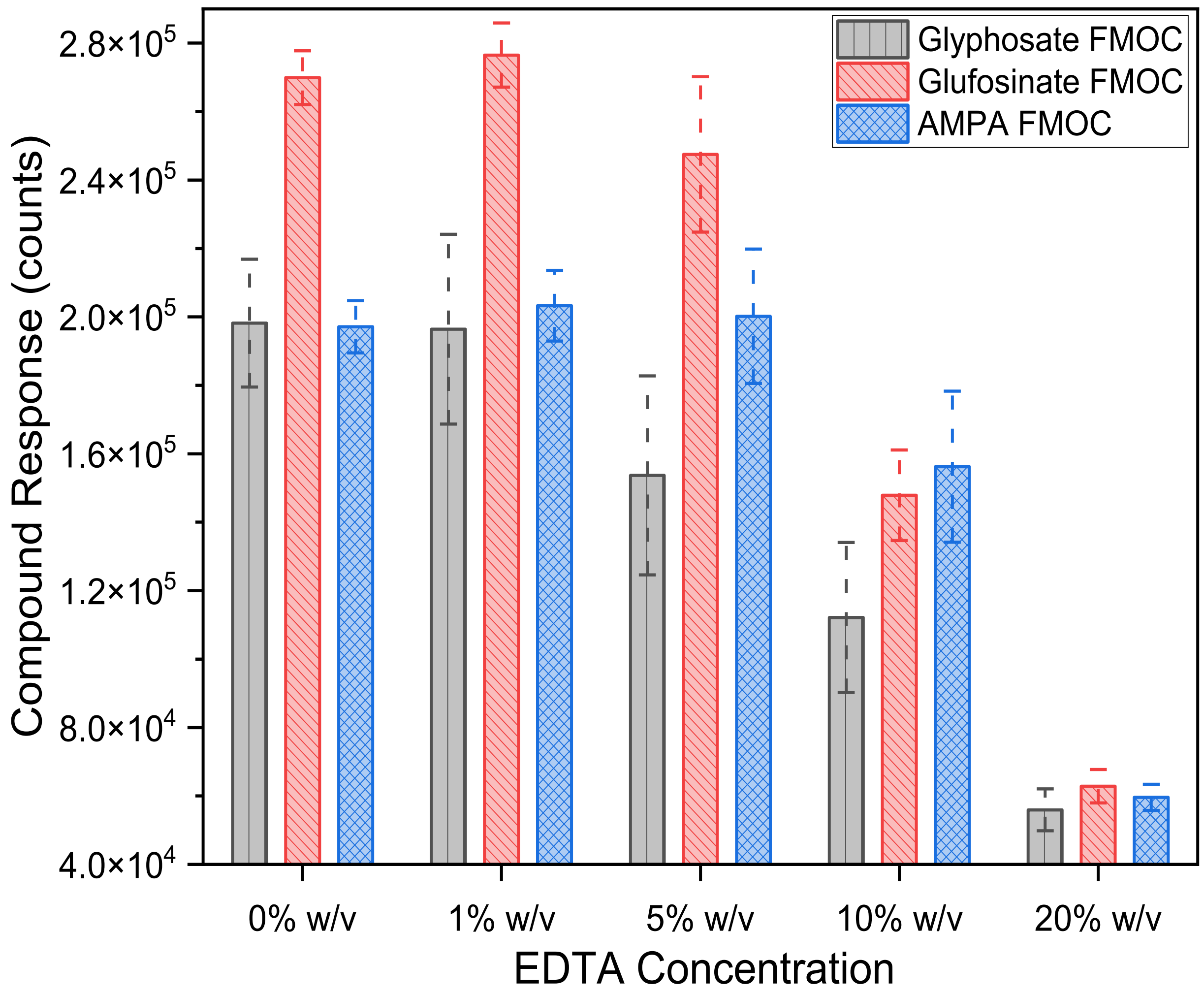

Supporting Information: Figure S3 - Chromatographic response of target compounds with varied EDTA concentrations (n=3, error bar = standard deviation).

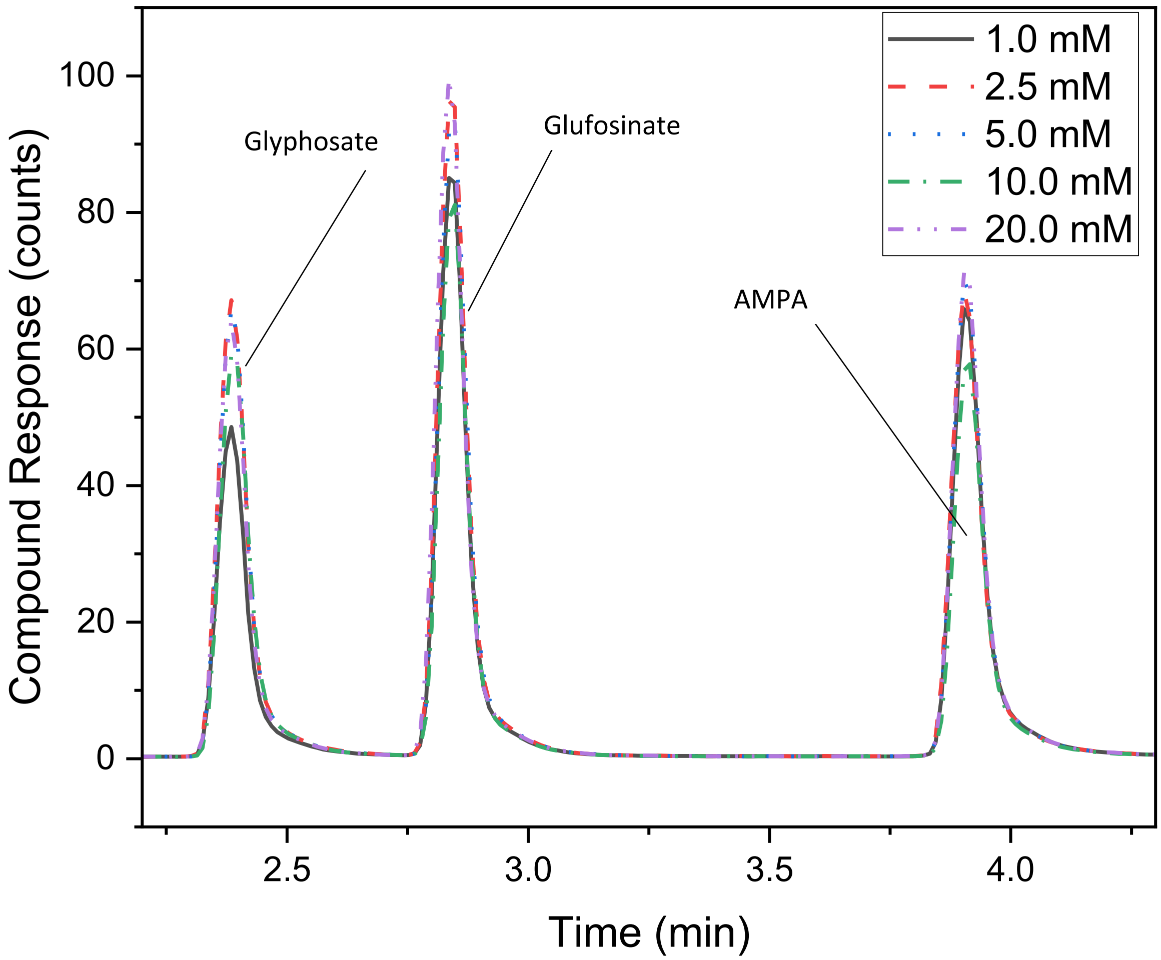

Supporting Information: Figure S4 - Chromatographic response of target compounds with varied FMOC concentrations.

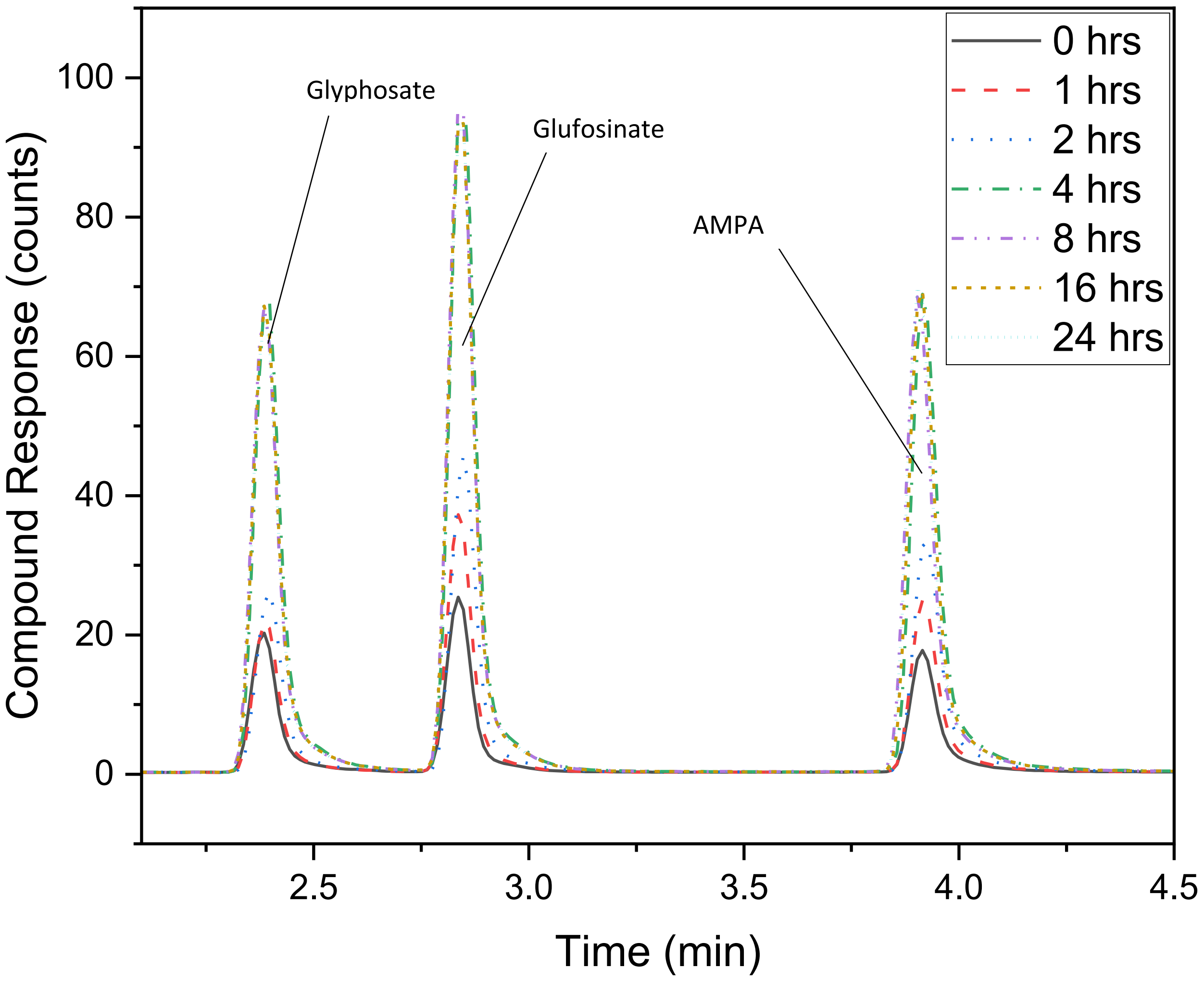

Supporting Information: Figure S5 - Chromatographic average response of target compounds with varied derivatization times.

Supporting Information: Table S1 - Desirability of optimal conditions for the complete derivatization of glyphosate, glufosinate, and AMPA in terms of reaction time and derivatizing agent concentration

**Borate (w/v)**	**FMOC-Cl (mM)**	**Derivatization time (hrs)**	**EDTA (w/v)**	**Total Desirability**	**Glyphosate Desirability**	**Glufosinate Desirability**	**AMPA Desirability**
5%	2.5	4	1%	**0.93**	0.96	0.87	0.95
5%	2.5	4	5%	**0.88**	0.85	0.85	0.92
5%	2.5	24	1%	**0.65**	0.63	0.56	0.76
5%	2.5	24	5%	**0.65**	0.59	0.58	0.78
5%	20	4	1%	**0.31**	0.41	0.22	0.33
5%	20	4	5%	**0.20**	0.23	0.14	0.23
5%	20	24	1%	**0.25**	0.29	0.24	0.22
5%	20	24	5%	**0.18**	0.17	0.19	0.17
10%	2.5	4	1%	**0.86**	0.85	0.81	0.92
10%	2.5	4	5%	**0.86**	0.83	0.84	0.92
10%	2.5	24	1%	**0.59**	0.57	0.50	0.72
10%	2.5	24	5%	**0.65**	0.61	0.58	0.79
10%	20	4	1%	**0.31**	0.38	0.24	0.34
10%	20	4	5%	**0.25**	0.28	0.20	0.27
10%	20	24	1%	**0.26**	0.31	0.25	0.22
10%	20	24	5%	**0.24**	0.27	0.26	0.21

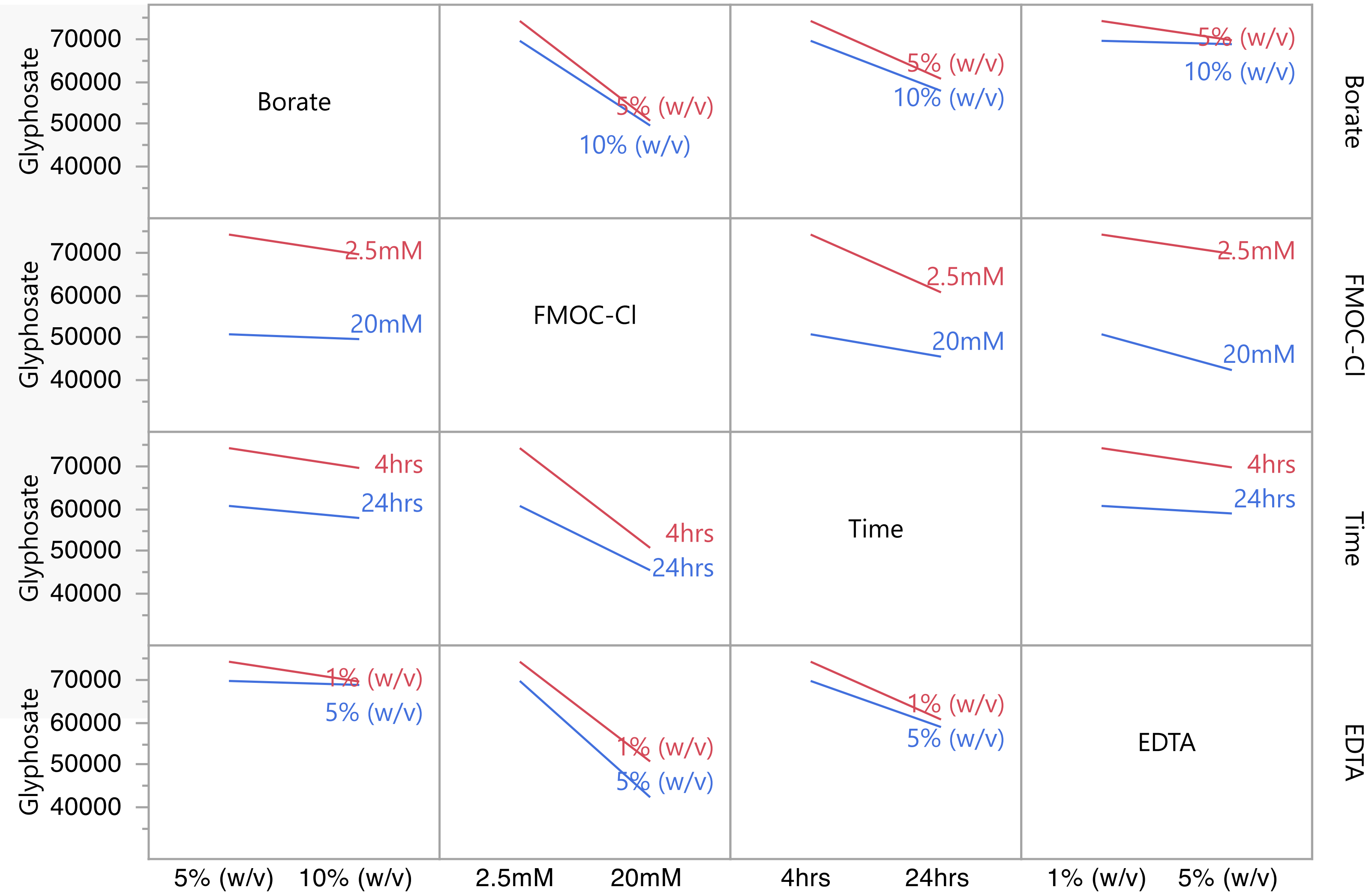

Supporting Information: Figure S6a - Interaction profile of the various derivatization factors for glyphosate.

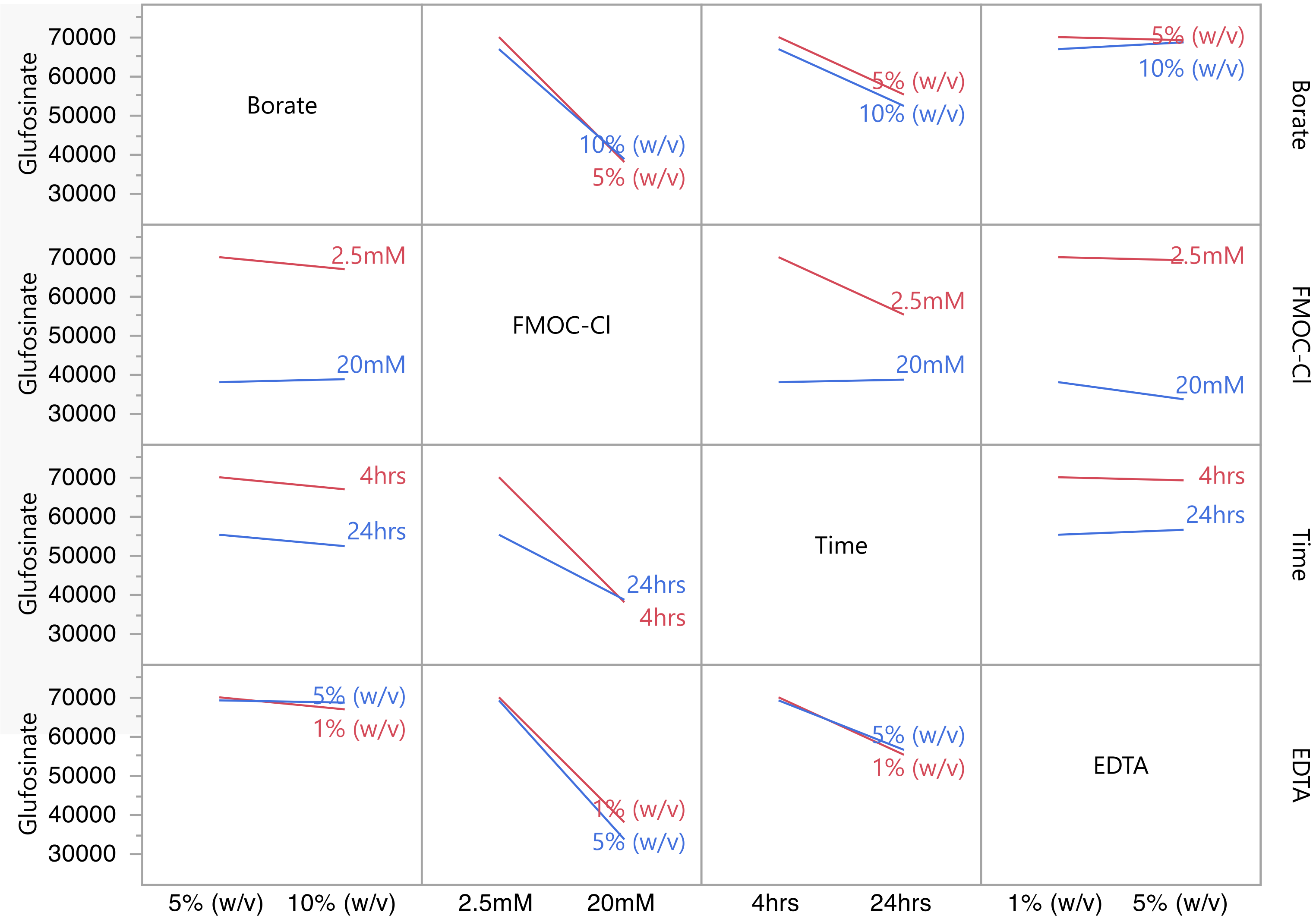

Supporting Information: Figure S6b - Interaction profile of the various derivatization factors for Glufosinate.

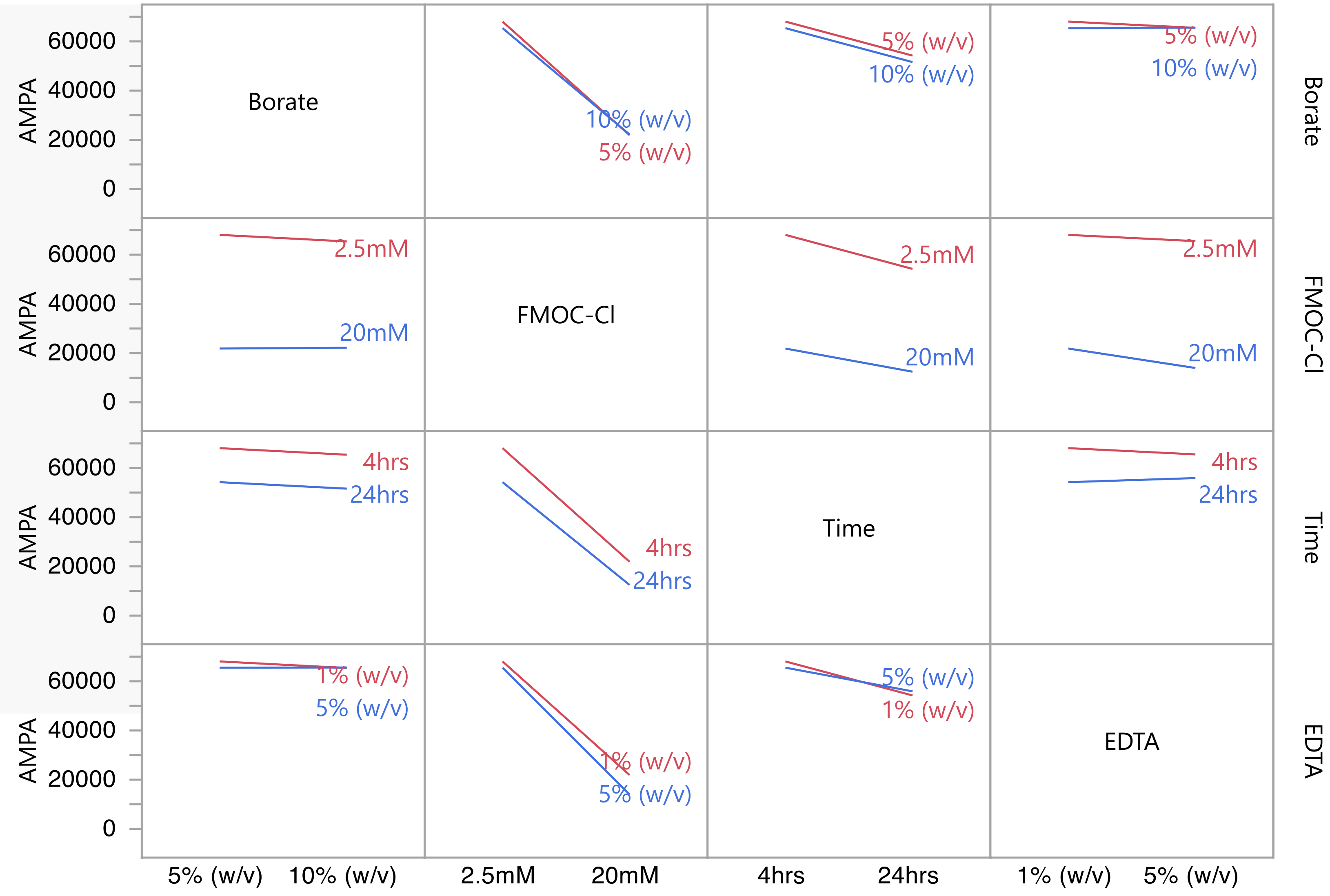

Supporting Information: Figure S6c - Interaction profile of the various derivatization factors for AMPA.

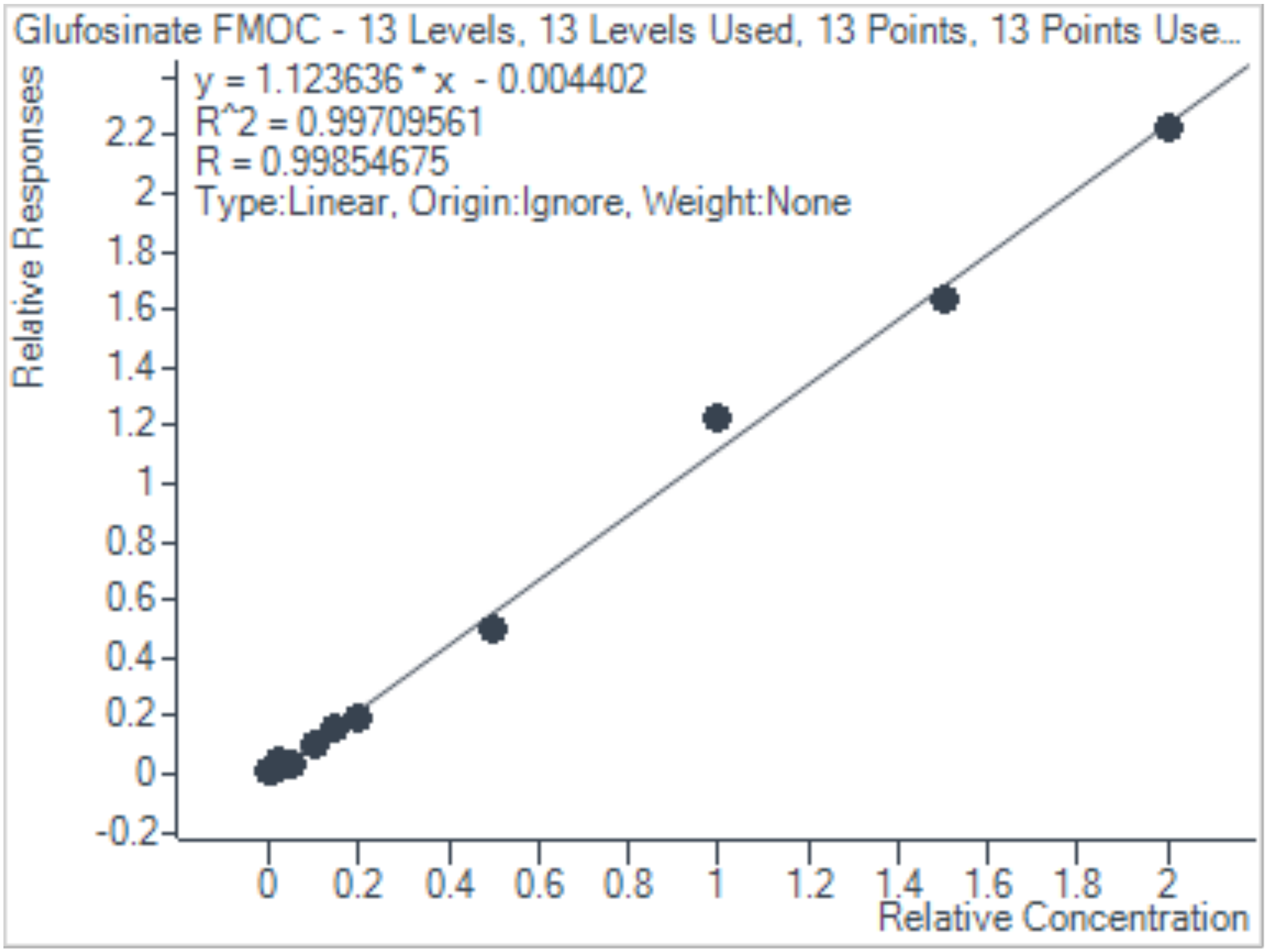

Supporting Information: Figure S7 - Calibration curve for Glufosinate (low level)

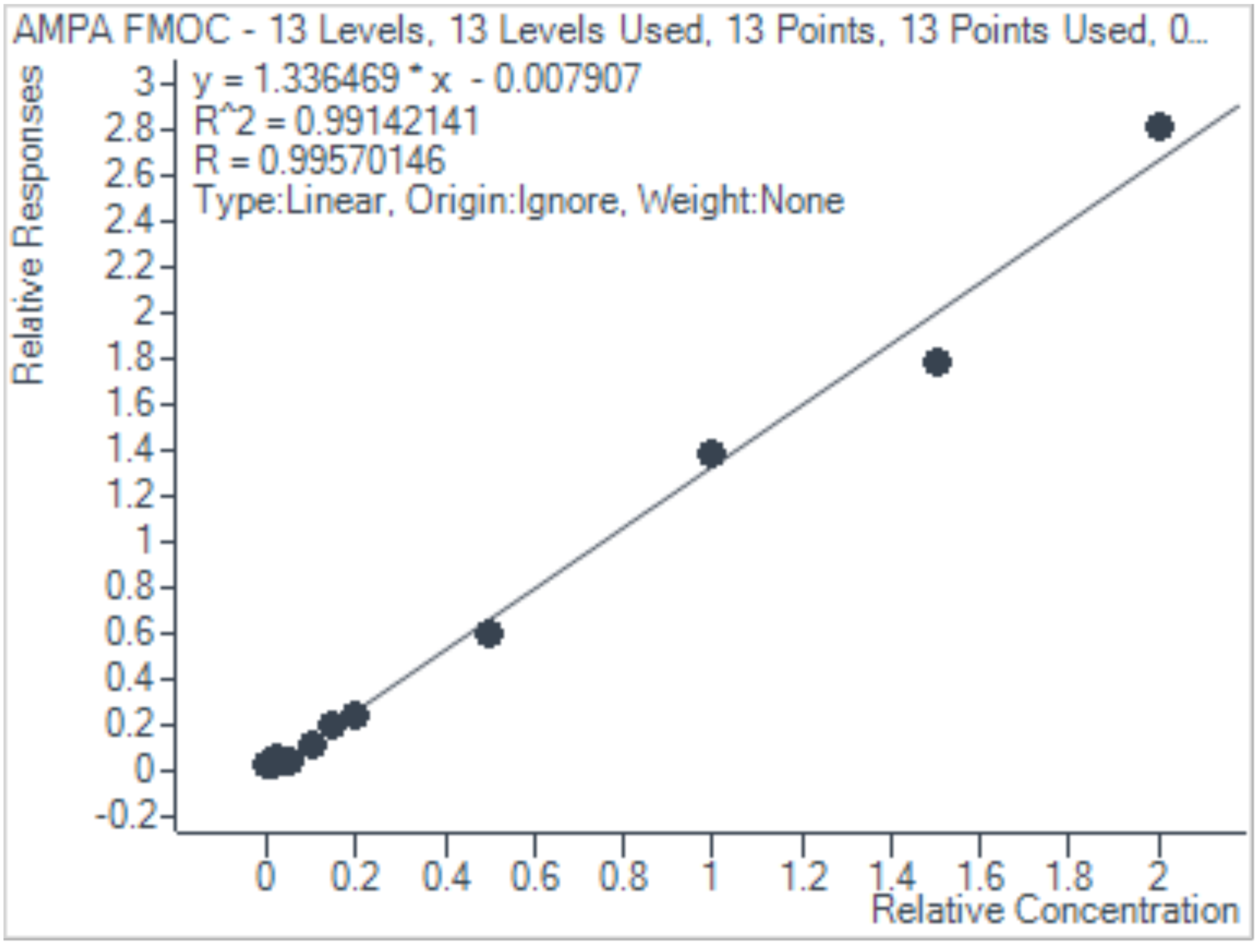

Supporting Information: Figure S8 - Calibration curve for AMPA (low level)

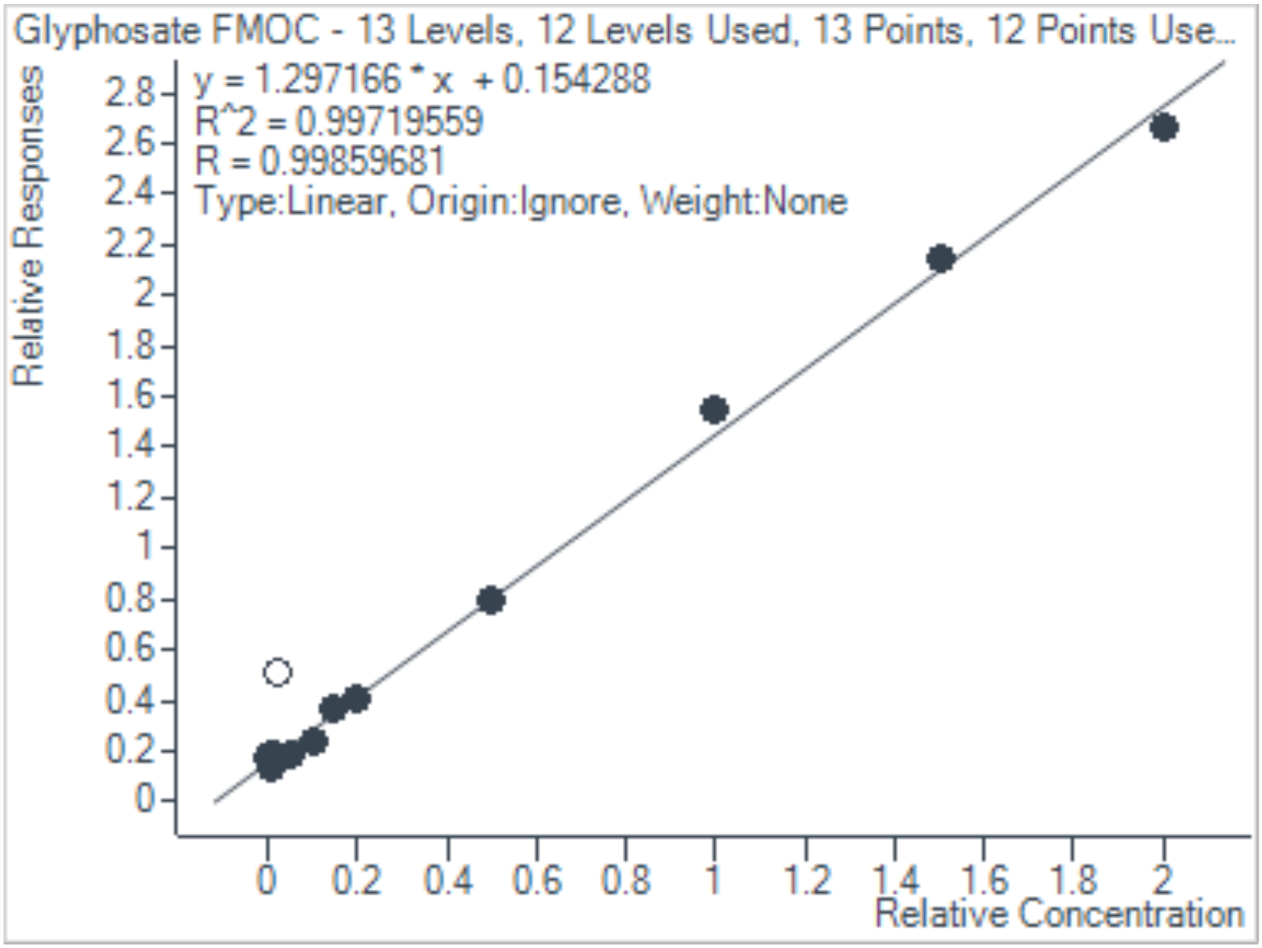

Supporting Information: Figure S9 - Calibration curve for Glyphosate (low level)

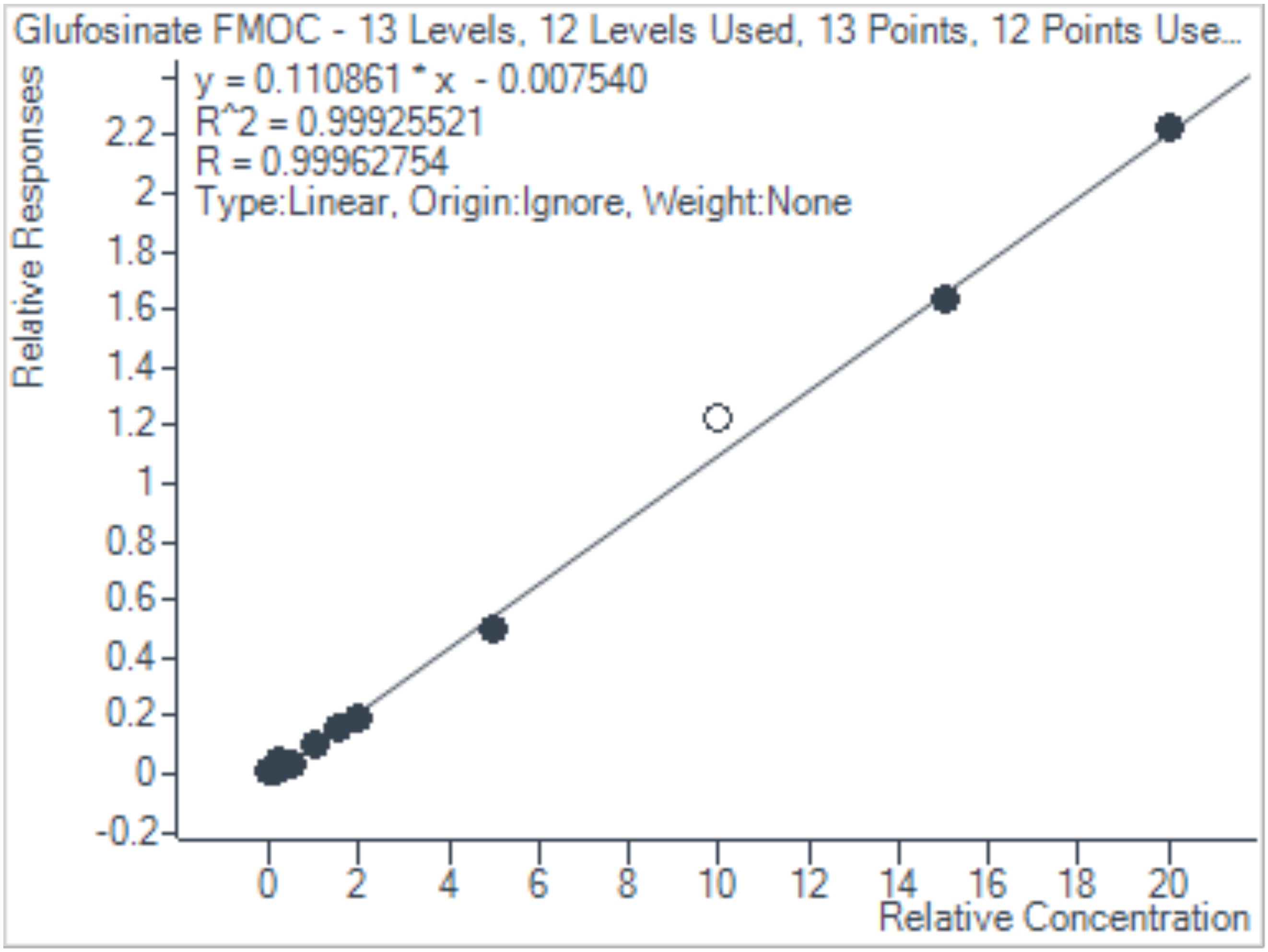

Supporting Information: Figure S10 - Calibration curve for Glufosinate (high level)

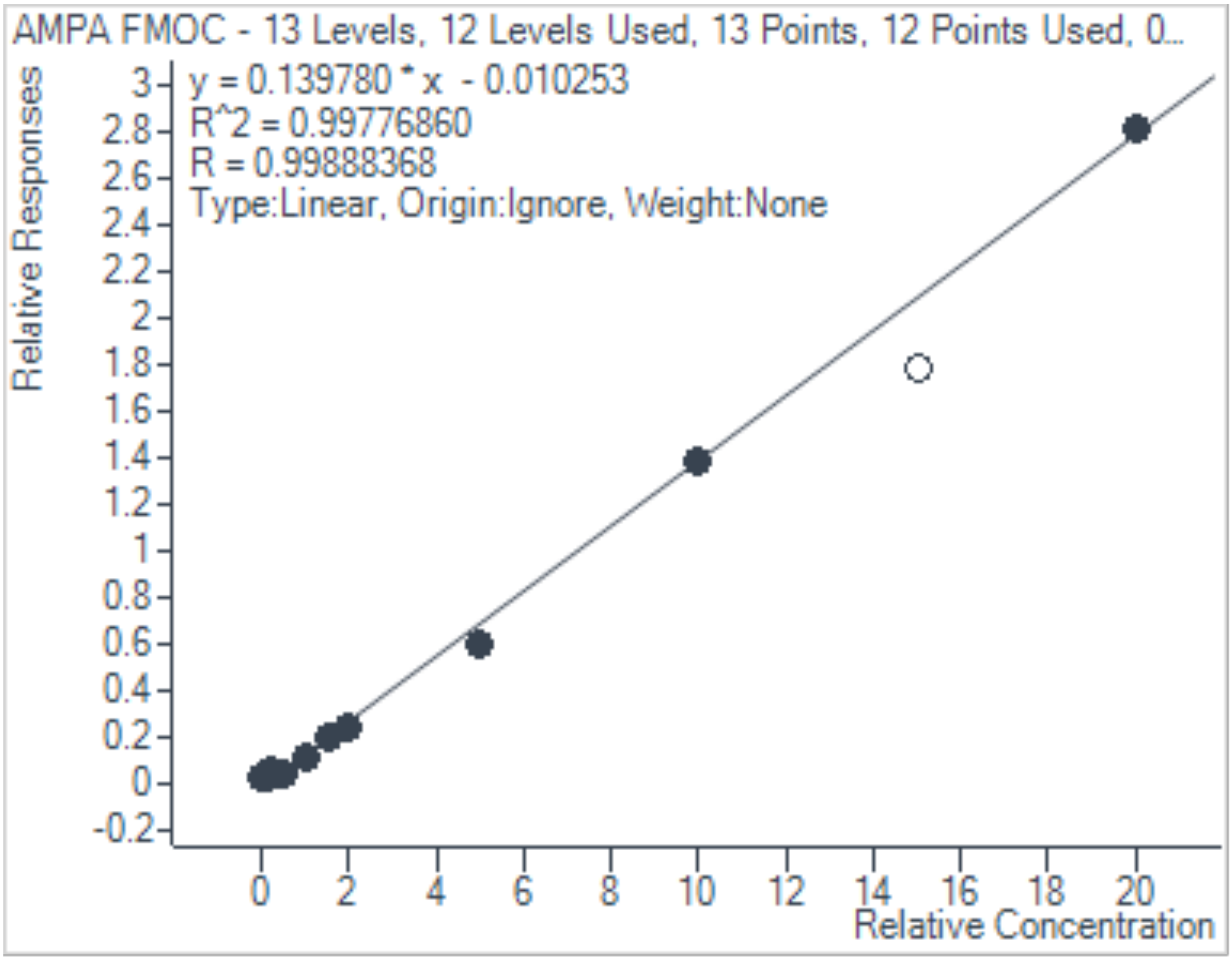

Supporting Information: Figure S11 - Calibration curve for AMPA (high level)

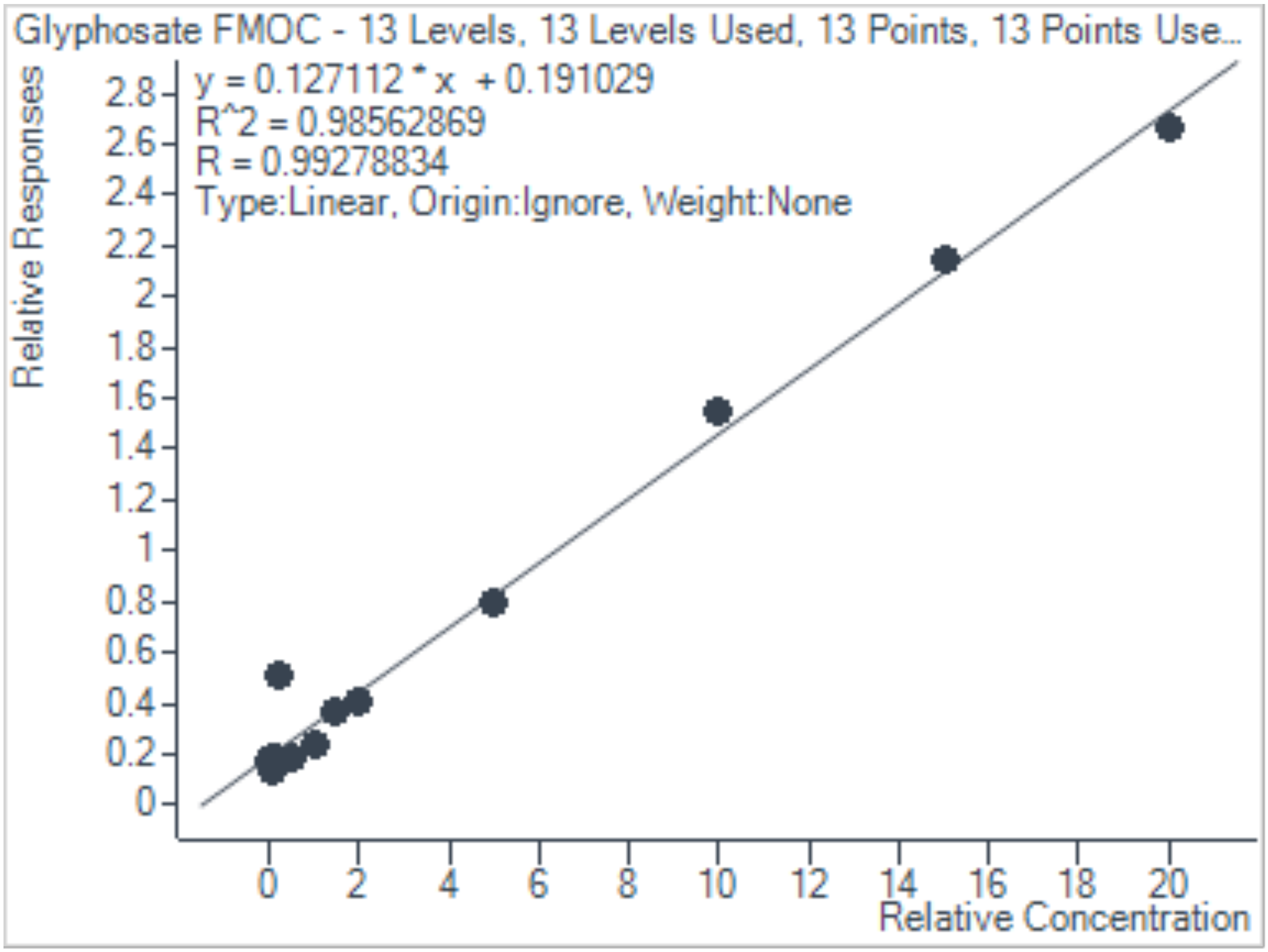

Supporting Information: Figure S12 - Calibration curve for Glyphosate (high level)
